# Smartphone Ownership Among Cardiology Inpatients Pre–COVID-19 and Post–COVID-19 Pandemic: Secondary Analysis of Randomized Controlled Trials

**DOI:** 10.2196/77141

**Published:** 2026-06-02

**Authors:** Sarah Badger, Praveen Indraratna, Jennifer Yu, James McVeigh, Joseph Magdy, Joan Li, Nigel Lovell, Sze-Yuan Ooi

**Affiliations:** 1 Department of Cardiology, Prince of Wales Hospital, Barker St, Randwick, Australia, 61 93822222; 2 School of Clinical Medicine, UNSW Sydney, Sydney, Australia; 3 School of Public Health and Community Medicine, UNSW Sydney, Sydney, Australia

**Keywords:** smartphone ownership, heart failure, coronary artery disease, mobile health, cardiac rehabilitation

## Abstract

**Background:**

Mobile health (mHealth) interventions are increasing in popularity for the management of heart failure and coronary artery disease. The use of these interventions is dependent on rates of smartphone ownership. It is estimated that approximately 90% of the Australian adult population owns a smartphone; however, international studies suggest that smartphone ownership is significantly lower in patient populations, ranging from 34% to 91%. Smartphone ownership in patients with cardiovascular disease has not previously been examined.

**Objective:**

This study aimed to examine and compare pre–COVID-19 and post–COVID-19 pandemic smartphone ownership rates of inpatients admitted with coronary artery disease or heart failure.

**Methods:**

Data from prescreening logs of 2 multicenter randomized controlled trials, TeleClinical Care (TCC)-Pilot and TCC-Cardiac, were reviewed. TCC-Pilot recruited patients between February 2019 and March 2020. This formed the pre–COVID-19 cohort, with 377 patients screened who lived in Sydney, had a qualifying hospital admission, and had information regarding their phone ownership status. TCC-Cardiac recruited patients from July 2021 to February 2023, with 718 patients meeting the criteria and forming the post–COVID-19 cohort. Supplemental patient demographic and medical history data were collected from the electronic medical record.

**Results:**

In the pre–COVID-19 cohort (N=377), 194 (51.5%) patients owned smartphones, 79 (21%) owned phones that were incompatible with the mHealth intervention, and the remaining 104 (27.6%) did not own a mobile phone. Smartphone owners were predominantly male (*P*<.001) and more often had private health insurance (*P*=.002). In the post–COVID-19 cohort (N=718), 366 (51%) patients owned smartphones, 106 (14.8%) owned incompatible phones, and the remaining 246 (34.3%) did not own any mobile phone. In both cohorts, younger patients were more likely to own smartphones (*P*<.001). Multiple comorbidities were associated with not owning a phone.

**Conclusions:**

Smartphone ownership accounted for just over 50% of the patients in this population. It was less common among older adults, patients with comorbidities, and those with markers of lower socioeconomic status. This needs to be considered when delivering mHealth interventions.

## Introduction

Cardiovascular disease is the leading cause of morbidity and mortality in Australia, accounting for 27% of deaths and 5.2% of all hospitalizations [1]. Heart failure (HF) and coronary artery disease (CAD) account for a significant proportion of hospitalizations, comprising 1.6% and 1.4%, respectively. Readmission rates of up to 20% for patients with CAD and HF in the 30 days after discharge have been observed [2-4], which is associated with increased mortality and substantial health care costs [2]. Many of these readmissions are preventable, with an audit of 3 hospitals in New South Wales finding that 27% of angina pectoris admissions and 63% of HF admissions could be prevented through effective and timely outpatient care [5]. Traditional cardiac rehabilitation (CR) reduces morbidity and cardiovascular mortality, reduces hospital admissions, and improves quality of life [6,7]. However, CR is poorly attended, with only 20% to 30% of eligible participants attending [8-10].

The use of mobile health (mHealth) interventions to reduce readmission rates and improve CR adherence is increasing following evidence of its benefit. A meta-analysis of patients with cardiovascular disease found a significant improvement in blood pressure and HF hospitalization rates with the use of mHealth interventions [11]. A separate systematic review of 17 studies, including 11 randomized controlled trials (RCTs), found that mHealth use in HF significantly improved quality of life, and showed a positive trend in quality of self-care, number and duration of hospitalizations, and medication adherence [12]. Other benefits of mHealth include improvements in uptake, adherence, and completion of CR [13,14], reduced HF mortality [15,16], and improved daily physical activity [17] and health literacy [18].

The use of mHealth interventions is dependent upon rates of smartphone ownership among the target population; thus, an understanding of this demographic is crucial in developing interventions. It is estimated that approximately 90% of the Australian adult population owns a smartphone, with 81% using the internet daily on their smartphone [19]. However, this is not a reliable indicator of smartphone use in patients with cardiovascular disease, who are typically older than the populations surveyed by the telecommunications industry. A total of 5 studies from 2019 and 2020 identified smartphone ownership rates in patient populations to be between 34% and 91%, with 3 of those studies examining populations in the United States [20-24]. Although the sample size was considerable, ranging from 146 to 555 patients, the median ages were low at less than 49 years for patients in the emergency department and inpatients with schizophrenia, and 62 years for patients in oncology and diabetes clinics. The median age for the 5th study was not reported. The highest smartphone ownership was in patients in the emergency department, and the lowest being in an inpatient psychiatric setting. To our knowledge, smartphone ownership in the cardiac patient population is unknown. Furthermore, the impact of COVID-19 on smartphone ownership is not known.

The aim of the proposed study is to examine and compare pre–COVID-19 and post–COVID-19 pandemic smartphone ownership rates of inpatients admitted with CAD or HF and determine factors that limit smartphone ownership. This would allow an exploration of the patient groups for which mHealth interventions may be unavailable, so alternative methods, including provision of smartphones or tablets, could be considered. Due to the lockdowns and social distancing during the COVID-19 pandemic, we postulate that a greater number of people gained access to smartphones as a social outlet during periods of isolation to attend telehealth appointments and for contact tracing or vaccination requirements. We propose that those with chronic health conditions who were at higher risk of severe COVID-19 would have been more affected by the prevention methods during the COVID-19 pandemic, and thus more likely to acquire a smartphone.

## Methods

This study examined prescreening data from 2 multicenter RCTs. The RCTs were conducted 15 months apart due to a New South Wales Health mandate requiring the suspension of all research during the height of the COVID-19 pandemic.

### TeleClinical Care

The initial pilot study, TeleClinical Care (TCC)-Pilot (ACTRN12618001547235), recruited patients between February 2019 and March 2020 from 2 hospitals in Sydney, New South Wales [25].

Patients were eligible if they were being discharged after an admission for either HF or acute coronary syndrome, were aged ≥18 years, and owned a smartphone. Patients were excluded if they were unable or unwilling to provide informed consent, unable to operate the app or attend in-person follow-up, or traveled overseas in the first 30 days after discharge. Patients were either randomized to usual care or TCC plus usual care. The intervention arm involved measuring blood pressure, heart rate, and weight daily to input into the TCC app on their smartphone, as well as wearing a fitness band which tracked minutes of activity per day. If an input was outside of the defined limits, an alert was emailed to the monitoring team (a cardiologist and a cardiac nurse practitioner) who reviewed the alert and contacted the patient, and if deemed necessary, their general practitioner or cardiologist would alter management. The primary objective was to examine the efficacy of the TCC model compared with usual care on the incidence of 30-day hospital readmission rates.

### TeleClinical Care-Cardiac

**Figure 1.**
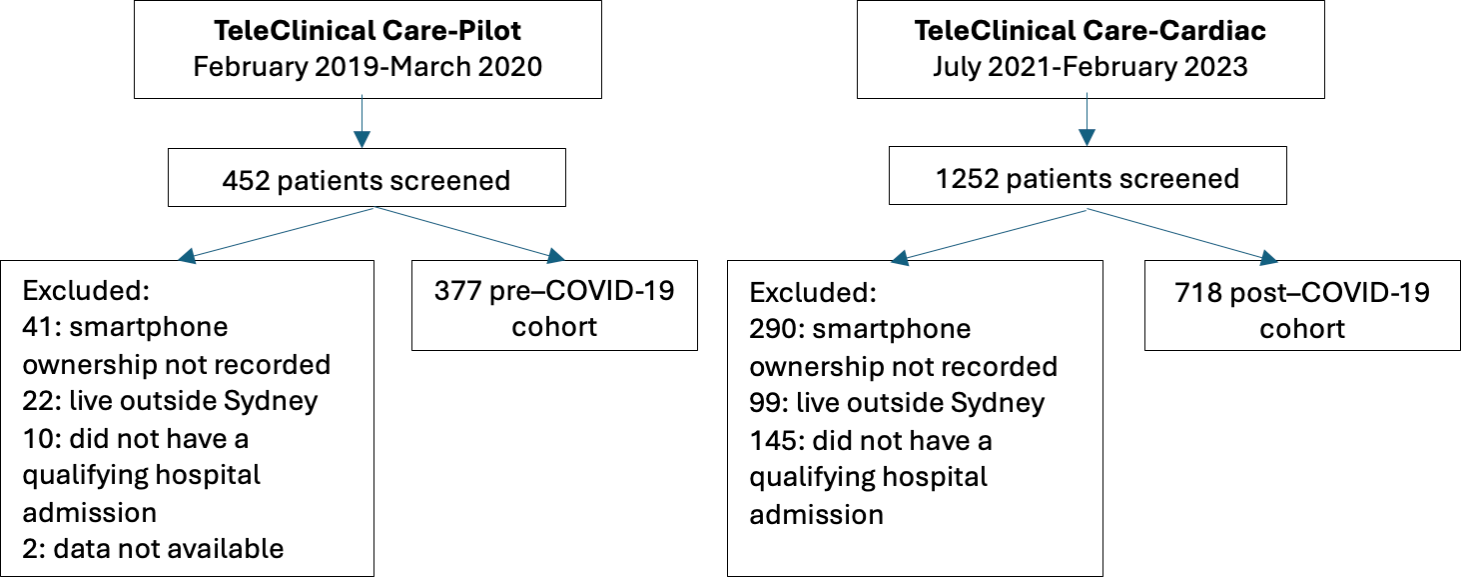
Flow diagram of patient screening and cohort selection for the TeleClinical Care-Pilot and TeleClinical Care-Cardiac cohorts.

The TCC-Cardiac RCT (ACTRN12621000754842) began recruitment in July 2021 from 8 public hospitals across regional and metropolitan New South Wales [26]. Patients were eligible if they were being discharged after an admission with acute myocardial infarction (AMI) or decompensated HF, provided written consent, and were aged ≥18 years. They were excluded if they had a cognitive impairment, terminal illness, limited English to operate the app or communicate with staff, enrolled in another active study, or were determined to not be able to adhere to study requirements. Patients were stratified into 1 of 3 cohorts depending on their access to technology. Cohort 1 were randomized to receive either the TCC-Cardiac model of care or usual care, cohort 2 participants were randomized to TCC-text or usual care, and cohort 3 was a registry of patients with no mobile phones receiving usual care. The TCC-Cardiac model of care collected the same data as the TCC intervention arm, as well as oxygen saturations. Additionally, the app provided medication reminders and measurement of compliance, a virtual exercise program, and education to reinforce health behaviors such as physical activity, smoking cessation, and healthy eating. The follow-up of this data was the same as the TCC intervention arm. The TCC-text intervention cohort received automated messages tailored to their diagnosis and smoking status with the aim to prompt positive behavioral change. The primary objective was the 6-month unplanned hospital readmission rate in cohort 1 patients randomized to TCC-Cardiac as an adjunct to usual care versus usual care alone. These RCTs form the basis of this study as outlined in Figure 1.

### Smartphone Screening Procedure

In both trials, screening involved visually inspecting phones to confirm it was an eligible smartphone, with a smartphone being defined as a mobile phone that has internet access and is capable of downloading, installing, and operating apps. If the patient did not have a compatible smartphone, then they were either categorized as having an incompatible phone (either unable to download or operate the TCC app, or a phone that does not have internet capability) or no phone.

### This Study

The TCC-Pilot data were grouped in the pre–COVID-19 cohort, while the TCC-Cardiac data were grouped in the post–COVID-19 cohort. Patients were excluded if smartphone ownership was not recorded, they did not live in Sydney, or they were not admitted secondary to HF or CAD. Data regarding smartphone ownership from TCC-Cardiac were used for this study until February 2023. Subsequently, supplemental patient demographic and medical history data were collected from the electronic medical record. The Socio-Economic Indexes for Areas Index of Relative Socio-Economic Advantage and Disadvantage was determined based on the patient’s postcode. Poor mobility was a clinical judgment based on their medical history, including high falls risk or assistance required to mobilize. Patient risks and comorbidities were compared according to phone ownership using the chi-square and Kruskal-Wallis tests for categorical and continuous variables, respectively. Logistic regression was used to estimate the odds ratios for smartphone ownership adjusted for age and sex. All analyses were conducted using Stata (version 15.1; StataCorp).

### Ethical Considerations

The study was reviewed and approved by the South-Eastern Sydney Local Health District Human Research Ethics Committee (2022/ETH01643). As this was a retrospective audit, consent was not required. All data were deidentified.

## Results

Mobile phone ownership was assessed in 377 patients in the pre–COVID-19 cohort and 718 patients in the post–COVID-19 cohort. Demographics and comorbidities of the patients in each cohort are described in Table 1. The 2 cohorts were similar with respect to age and sex. Statistical differences were seen in some comorbidities, including primary diagnosis (*P*=.002), hypertension (*P*=.03), peripheral arterial disease (*P*=.01), cerebrovascular disease (*P*=.02), hypercholesterolemia (*P*<.001), and chronic kidney disease (*P*=.002). Smartphone ownership was similar in both cohorts, but a shift was noted from incompatible mobile phones to having no phone in the latter period (*P*=.04). In the pre–COVID-19 cohort, 194 (51.5%) patients owned smartphones, 79 (21.2%) owned incompatible phones, and the remaining 104 (27.6%) did not own any mobile phone. In the post–COVID-19 cohort, 366 (50.9%) patients owned smartphones, 106 (14.8%) owned incompatible phones, and the remaining 246 (34.3%) did not own any mobile phone.

**Table 1.** Comparison of the demographics and phone status of the pre–COVID-19 and post–COVID-19 cohorts.

	Pre–COVID-19 (n=377)	Post–COVID-19 (n=718)	*P* value
Age (years), median (IQR)	73 (61‐83)	74 (64‐83)	.18
Gender, n (%)			.24
Man	240 (63.7)	489 (68.1)	
Woman	137 (36.3)	228 (31.8)	
Nonbinary	0 (0)	1 (0.1)	
Aboriginal and/or Torres Strait Islander, n (%)	8 (2.1)	13 (1.8)	.50
Born in Australia, n (%)	171 (45.7)	324 (45.2)	.87
Private health insurance, n (%)	170 (45.2)	285 (40.2)	.03
SEIFA IRSAD^a^ (decile), <9, n (%)	9 (2.4)	32 (4.5)	.36
SEIFA IRSAD^a^ (decile), 9, n (%)	212 (56.2)	389 (54.2)	.36
SEIFA IRSAD^a^ (decile), 10, n (%)	156 (41.4)	297 (41.4)	.36
Primary diagnosis, n (%)			.002
Acute myocardial infarction	188 (49.9)	298 (41.5)	
Unstable angina	15 (4)	14 (1.9)	
Decompensated heart failure	174 (46.2)	406 (56.5)	
Past medical history, n (%)			
Coronary artery disease	147 (39.3)	240 (33.7)	.07
Myocardial infarction	114 (30.5)	184 (25.8)	.10
Percutaneous coronary intervention	78 (20.9)	150 (21.3)	.87
Coronary artery bypass grafting	53 (14.2)	75 (10.5)	.08
Heart failure	104 (27.8)	230 (32.5)	.11
Atrial fibrillation	106 (28.3)	227 (31.9)	.22
Ventricular tachycardia	11 (3.0)	11 (1.5)	.12
Valvular disease	36 (9.6)	71 (10.1)	.81
Valvular surgery	22 (6.0)	51 (7.2)	.45
Hypertension	242 (64.2)	408 (57.3)	.03
Hypercholesterolaemia	209 (55.7)	320 (45.1)	<.001
Diabetes mellitus			.85
Type 1	7 (1.9)	13 (1.8)	
Type 2	116 (31.4)	233 (33.1)	
Chronic lung disease	50 (13.6)	86 (12.1)	.48
Chronic kidney disease	93 (24.9)	119 (17)	.002
Smoking history			.09
Current smoker	65 (17.6)	115 (17)	
Ex-smoker	116 (31.4)	173 (25.6)	
Peripheral arterial disease	43 (11.5)	48 (6.8)	.01
Cerebrovascular disease	33 (8.8)	36 (5.1)	.02
Stroke	20 (5.3)	50 (7)	.28
Transient ischemic attack	5 (1.3)	12 (1.7)	.65
Malignancy	16 (4.3)	48 (6.7)	.10
Deep vein thrombosis or pulmonary embolus	15 (4.0)	33 (4.6)	.63
Chronic liver disease	2 (0.5)	11 (1.5)	.14
Gastroesophageal reflux disease	52 (13.9)	110 (15.4)	.49
Poor mobility	61 (16.3)	126 (17.9)	.51
Phone status, n (%)			.04
Compatible smartphone	194 (51.5)	366 (51.0)	
Incompatible phone	79 (21.0)	106 (14.8)	
No phone	104 (27.6)	246 (34.3)	

a SEIFA IRSAD: Socio-Economic Indexes for Areas Index of Relative Socio-Economic Advantage and Disadvantage.

In the pre–COVID-19 cohort, the median age of the participants was 73 (IQR 61‐83) years. A total of 240 (63.7%) participants were male and 170 (45.2%) had private health insurance. Notably, private health insurance is used as a surrogate of wealth, given free health care through Medicare is available to all citizens in Australia. This is similar to the post–COVID-19 cohort, in which the average age of the participants was 74 years (IQR 64‐83). A total of 489 (68.1%) participants were male, 13 (1.8%) identified as Aboriginal and/or Torres Strait Islander, 324 (45.2%) were born in Australia, and 285 (40.2%) had private health insurance.

In the prepandemic period, smartphone owners were predominantly male (*P*<.001) and more often had private health insurance (*P*=.002). In both cohorts, younger patients were more likely to own smartphones (*P*<.001; Table 2).

**Table 2.** Age, gender, and private health insurance stratified by phone ownership in the pre–COVID-19 and post–COVID-19 cohort, identifying the significance of phone ownership by subgroup.

Variable	Smartphone	Incompatible phone	No phone	*P* value
Pre–COVID-19				
Age (years; n=377), median (IQR)	63 (55-73)	76 (69-84)	83 (77-90)	<.001
Age (years), n (%)				<.001
<49 (n=28)	27 (96.4)	1 (3.6)	0 (0)	
50‐59 (n=51)	39 (76.5)	7 (13.7)	5 (9.8)	
60‐69 (n=80)	62 (77.5)	12 (15.0)	6 (7.5)	
70‐79 (n=94)	42 (44.7)	27 (28.7)	25 (26.6)	
>80 (n=124)	24 (19.4)	32 (25.8)	68 (54.8)	
Gender, n (%)				<.001
Man (n=240)	147 (61.3)	41 (17.1)	52 (21.7)	
Woman (n=137)	47 (34.3)	38 (27.7)	52 (38.0)	
Private health insurance, n (%)				.002
Yes (n=170)	103 (60.6)	24 (14.1)	43 (25.3)	
No (n=206)	91 (44.2)	54 (26.2)	61 (29.6)	
Post–COVID-19				
Age (years; n=718), median (IQR)	68 (55-77)	74.5 (69-83)	83 (75-89)	<.001
Age (years), n (%)				<.001
<49 (n=48)	43 (89.6)	5 (10.4)	0 (0)	
50‐59 (n=98)	84 (85.7)	9 (9.2)	5 (5.1)	
60‐69 (n=106)	80 (75.5)	13 (12.3)	13 (12.3)	
70‐79 (n=212)	97 (45.8)	42 (19.8)	73 (34.4)	
>80 (n=254)	62 (24.4)	37 (14.6)	155 (61.0)	
Gender, n (%)				.20
Man (n=489)	263 (53.8)	68 (13.9)	158 (32.3)	
Woman (n=228)	102 (44.7)	38 (16.7)	88 (38.6)	
Nonbinary (n=1)	1 (100)	0 (0)	0 (0)	
Private health insurance, n (%)				.27
Yes (n=285)	155 (54.4)	38 (13.3)	92 (32.3)	
No (n=424)	205 (48.3)	68 (16.0)	151 (35.6)	

**Figure 2.**
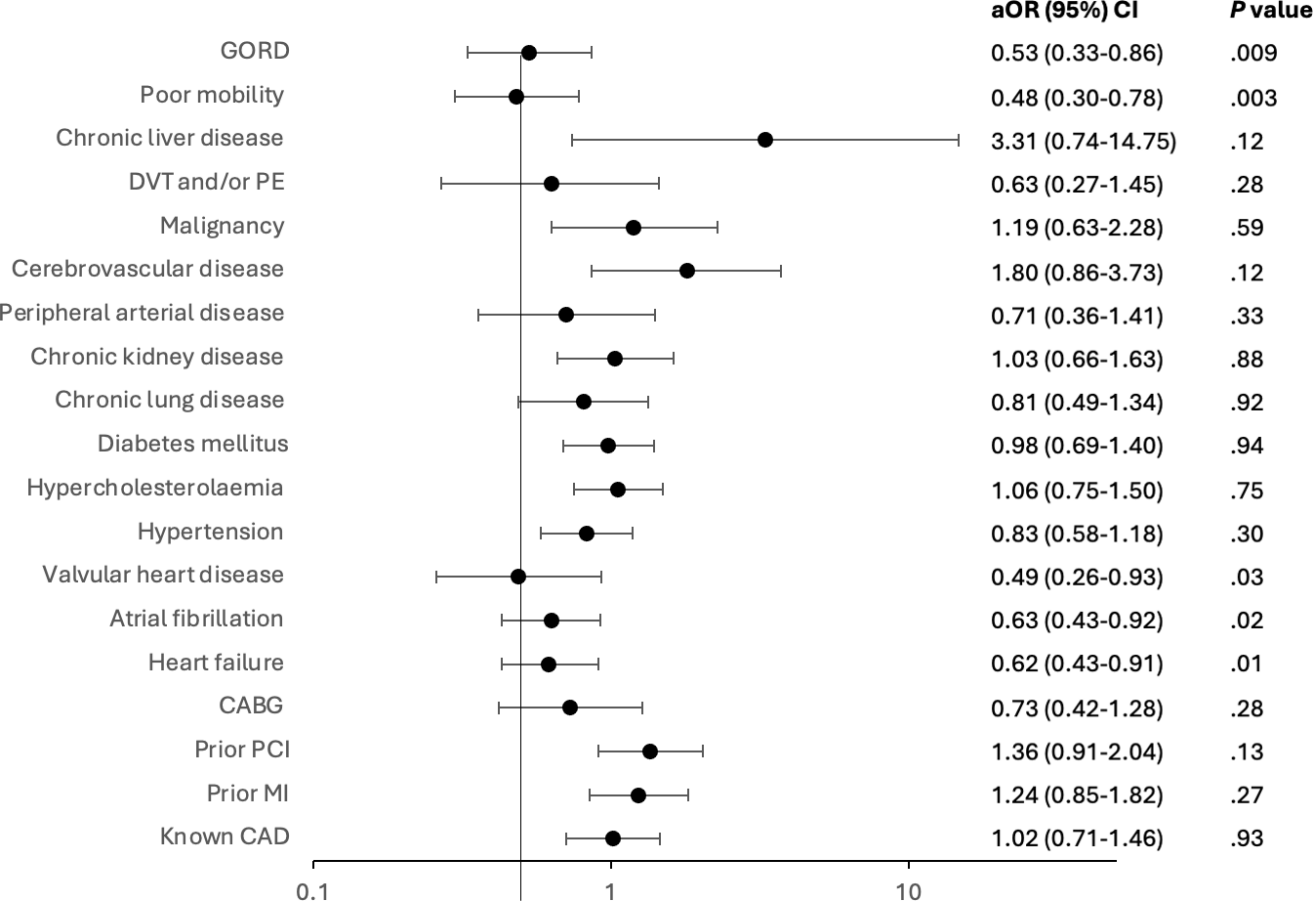
Relationship between smartphone ownership and comorbidities when adjusted for age and sex. Adjusted odds ratio (aOR) of smartphone versus no phone for comorbidities when controlled for age and sex. CABG: coronary artery bypass graft; CAD: coronary artery disease; DVT: deep vein thrombosis; GORD: gastroesophageal reflux disease; MI: myocardial infarction; PCI: percutaneous coronary intervention; PE: pulmonary embolus.

In the post–COVID-19 cohort, patients who had a history of CAD (*P*=.002), HF (*P*<.001), atrial fibrillation (*P*<.001), valvular heart disease (*P*<.001), hypertension (*P*<.001), hypercholesterolaemia (*P*=.004), chronic lung disease (*P*=.02), chronic kidney disease (*P*<.001), peripheral arterial disease (*P*<.001), and gastroesophageal reflux (GORD; *P*<.001) were significantly less likely to own a smartphone (Table 3). Poor mobility was also significantly associated with having no smartphone. When this was adjusted for age and sex, patients with a history of GORD, poor mobility, valvular heart disease, atrial fibrillation, and HF were significantly less likely to own a smartphone (Figure 2).

**Table 3.** Association between comorbidities and phone ownership in the post–COVID-19 cohort, identifying the significance of phone ownership by prior diagnoses (N=718).

	Smartphone (n=366), n (%)	Incompatible phone (n=106), n (%)	No phone (n=246), n (%)	*P* value
Known CAD^a^ (n=240)	100 (42)	40 (17)	100 (42)	.002
Prior AMI^b^ (n=184)	82 (45)	28 (15)	74 (40)	.13
Prior PCI^c^ (n=150)	60 (44)	21 (14)	69 (46)	.41
CABG^d^ (n=75)	21 (29)	13 (16)	41 (55)	<.001
Heart failure (n=230)	75 (33)	38 (17)	117 (51)	<.001
Atrial fibrillation (n=227)	68 (30)	36 (16)	123 (54)	<.001
Valvular heart disease (n=71)	19 (27)	14 (20)	38 (54)	<.001
Hypertension (n=408)	180 (44)	61 (15)	167 (41)	<.001
Hypercholesterolemia (n=320)	141 (44)	54 (17)	125 (39)	.004
Diabetes mellitus (n=233)	108 (46)	32 (14)	93 (40)	.07
Chronic lung disease (n=86)	33 (38)	12 (14)	41 (48)	.02
Chronic kidney disease (n=119)	43 (36)	16 (14)	60 (50)	<.001
Peripheral arterial disease (n=48)	14 (29)	5 (10)	29 (60)	<.001
Cerebrovascular disease (n=36)	17 (47)	4 (11)	15 (42)	.61
Malignancy (n=48)	20 (42)	9 (19)	19 (40)	.41
DVT^e^ or PE^f^ (n=33)	11 (33)	6 (18)	16 (48)	.20
Chronic liver disease (n=11)	8 (73)	2 (18)	1 (9)	.20
GORD^g^ (n=110)	35 (32)	22 (20)	53 (48)	<.001
Poor mobility (n=126)	29 (24)	23 (18)	74 (58)	<.001

a CAD: coronary artery disease.

b AMI: acute myocardial infarction.

c PCI: percutaneous coronary intervention.

d CABG: coronary artery bypass graft.

e DVT: deep vein thrombosis.

f PE: pulmonary embolism.

g GORD: gastroesophageal reflux disease.

## Discussion

### Principal Findings

This study is currently the largest study of smartphone ownership in an inpatient population. In this population of patients with AMI, unstable angina, and decompensated HF, smartphone ownership was just over 50%. It was less common among older adults; patients with comorbidities; and those with markers of lower socioeconomic status, such as lack of private health insurance. This study highlights that mHealth interventions could fail to address the health care disparities they set out to overcome and are likely excluding the most vulnerable population. Understanding smartphone ownership patterns is vital to tailoring mHealth interventions and improving accessibility.

### Comparison With Prior Work

This study is not alone in highlighting the inequity of mHealth interventions due to low smartphone ownership rates. Similarly, in a survey of 100 outpatients with HF in the United States, 93% owned a mobile phone, with only 68% owning a smartphone [27]. In contrast, in a 2018 survey of 50 US patients (mean age 64.5, SD 8.3 years; 32% women), the study by Sohn et al [28] found that 90% of patients with HF owned a smartphone and 49% owned a tablet. However, the study population consisted of mainly White, male patients aged 50 to 80 years, which is a younger and less diverse population than our own. More recent studies have also found varying results depending on geographical location and setting examined. A study of a low socioeconomic district in South Africa investigating mHealth interventions for HIV found that of the 788 individuals surveyed, 86% owned a personal mobile phone but only 43% of these were smartphones [29]. A study of 949 dialysis patients in the United States found that 81% owned smartphones or other internet-capable devices, with 72% using the internet [30]. Rates of smartphone ownership vary significantly between patient populations; however, the trend has not been increasing in recent years when comparing studies. Our hypothesis that the COVID-19 pandemic would increase smartphone ownership appears to be false in the target population; however, the collected data present a valuable insight into smartphone ownership patterns in the surveyed population.

This is contrasted with studies looking at smartphone ownership in the general population. The Health Information National Trends Survey, a nationally representative cross-sectional survey of civilian, noninstitutionalized adults aged >18 years in the United States, found that of the 5438 responders, 87.8% were smartphone owners, 8.3% had incompatible mobile phones only, and 3.9% had no phone [31]. This cohort had equal male to female respondents with an average age of 49 years and 63.5% identifying as White. In a subset of this population who identified as having or being at risk for cardiovascular disease, there was a smaller but still high prevalence of smartphone ownership (73%) [32]. This is similar to the Pew Research Center [33] cross-sectional survey of 5626 people living in the United States which found that 98% of people owned a mobile phone, with 91% having smartphones. Similarly, the respondents to this survey were predominantly aged <65 years and White; information on gender was not collected. Clearly, our inpatient population is not reflective of the general population in terms of age, sex, or smartphone ownership. Drawing broadly from these studies in conjunction with our own confirms that inpatients have lower rates of smartphone ownership than the general population.

A deeper analysis of smartphone ownership patterns within the patient population is required to understand the potential reach of the current and future mHealth interventions, as well as to ensure equity in these interventions. Many studies agree that there is a digital divide between younger and older adult patients, with younger people being more likely to own a smartphone [27,29-32,34]. This is especially highlighted by the Pew Research Center [33] survey which found 98% of people aged 18 to 29 years owned smartphones, but this decreased to 79% in those aged >65 years. The study by Yao et al [35] highlights in their scoping review that socially disadvantaged groups have more challenges in accessing digital health technologies, which may lead to more severe health inequities. This is supported by multiple studies showing that people who are employed, female [29], and have higher levels of education are more likely to own a smartphone [30-32,36]. Our study supports this with higher rates of private health insurance, a marker of higher socioeconomic status, being associated with smartphone ownership. Greater health literacy has also been associated with smartphone ownership [34,37]. Our study is the first to identify a detailed list of conditions associated with lower smartphone ownership; specifically, a history of GORD, poor mobility, valvular heart disease, atrial fibrillation, and HF. It is proposed that having a history of GORD is associated with not owning a smartphone, given it is known to be correlated with low socioeconomic status. However, the study by Na and Sheu [31] identified that not owning a smartphone was associated with chronic conditions such as being deaf, heart disease, lung disease, and cancer.

mHealth is a useful tool for addressing health disparities through equitable access to health information; however, there remain health inequities caused by the adoption of digital health technologies in health care. The determinants of access to mHealth are complex and often interrelated. Given that smartphones are a relatively new technology, a significant barrier is the lack of perceived usefulness and ease of use of the technology system preventing patients from prioritizing smartphone ownership [38]. There are both technology factors, such as lack of network infrastructure particularly in regional areas (eg, power, network coverage, and internet access) and the availability of smartphones, as well as patient factors (eg, age, sex, race, rurality, educational attainment, income, poor health conditions, health literacy, and education level) that contribute to not owning a smartphone.

Smartphone ownership in older adults is the most well-researched factor. The study by Wildenbos et al [39] identified 4 domains that were barriers to mHealth uptake in this population: cognition, motivation, physical ability, and perception. Learning to use mobile technology can be a challenging task due to little prior knowledge [40], lack of confidence [41], and physical impairments such as poor vision and fine motor skills [42,43]. Using mobile technology for mHealth interventions is an added complexity as the individual requires digital health literacy, a complex competency that includes health literacy, digital literacy, and the ability to make decisions on the basis of digital health data [44]. The reasons for lack of smartphone ownership are many and varied; however, it is the first barrier to accessing mHealth interventions.

Given almost half of the target population cannot access mHealth interventions in this study, strategies need to be devised to increase access to these apps to reduce hospitalization rates [11]. The study by Yao et al [35] suggests government agencies and medical institutions should provide help with resources to improve accessibility of mHealth. The aim would be to recoup the cost of these devices by avoiding hospital admissions, with mobile phones and tablets being available for AUD $200 (US $142.51) and data plans for AUD $180 (US $128.26) per year, compared with the average cost of a hospital bed in New South Wales Health of AUD $1660 (US $1182.83) per day [45], with cardiology and coronary care beds exceeding this. This suggests that the cost of a mobile phone is diminutive in comparison to readmission, a hypothesis that should be evaluated in future research. Companies should be encouraged to donate tablets and smartphones to hospitals, and software developers should be encouraged to maintain backward compatibility of their apps with older versions of devices and mobile operating systems. This would improve the accessibility to such services for socially disadvantaged groups. If a low rate of smartphone ownership is present in a population, app-based interventions may not be feasible, and alternative methodologies may be more equitable such as telephone or SMS text message–based services. Once accessibility has been overcome, the design of the intervention needs to be curated with consideration of the target group, preferably with their input during the design and implementation stages [35,46]. This will maximize accessibility and acceptability for all groups who use the app, reducing disparities. To ensure perceived usefulness, this needs to be explicitly communicated to the patient during the recruitment phase. Finally, new users need to be adequately supported when adopting novel mHealth interventions, especially when there is evidence supporting their efficacy.

### Limitations

Although this is the largest study of smartphone ownership in inpatients, there are limitations. First, the study excluded patients with cognitive impairment or limited English proficiency. These patient populations often face the highest barriers to digital literacy and health care access, thus potentially underestimating the digital divide. Second, a significant number of patients were excluded from the post–COVID-19 cohort due to missing phone ownership details. The phone must have been visualized to be included in the study; this is beneficial as it ensures accuracy but also confounds the data as patients who did not bring their phone to hospital were excluded from the study. Third, it was difficult to assess the patients’ sociodemographics completely given employment status was not available. The participants were primarily located at 1 local health district in a relatively affluent area in Sydney as shown by a median decile Socio-Economic Indexes for Areas Index of Relative Socio-Economic Advantage and Disadvantage of 9 out of 10 in both cohorts. However, this tool does not account for the nuances of this area where social housing is spread among the wealthy. As such, private health insurance status was chosen to represent socioeconomic status as a reflection of discretionary spending. Fourth, the poor mobility metric was based on clinical judgment, which is subjective and may introduce bias. Fifth, as acknowledged, there are many factors affecting smartphone ownership; however, only age and sex were adjusted for. Finally, only patients with an admission diagnosis of HF, AMI, and unstable angina were included, and therefore we are unable to extrapolate to other patient populations.

### Conclusions

mHealth interventions were introduced to eliminate health disparities and have the potential to improve chronic disease management. However, inequality in smartphone ownership in the highest risk comorbid patients as seen in this cohort has the potential to exacerbate rather than eliminate these disparities. Interestingly, this study showed that there was no improvement in smartphone ownership during the COVID-19 pandemic. Until smartphone ownership is commonplace in patient populations, these groups will continue to be systemically excluded. Health care providers who plan to use mHealth interventions in their practice need to ensure accessibility to all and have contingency plans if participants do not own a smartphone, such as providing a phone or tablet, or using other means to provide the same service.

## Abbreviations

AMI: acute myocardial infarction
CAD: coronary artery disease
CR: cardiac rehabilitation
GORD: gastroesophagealreflux disease
HF: heart failure
mHealth: mobile health
RCT: randomized controlled trial
TCC: TeleClinical Care
